# Steps towards automated image segmentation as part of a 3D printing pipeline in congenital heart disease

**DOI:** 10.1186/1532-429X-18-S1-P190

**Published:** 2016-01-27

**Authors:** Nicholas Byrne, Mari-Nieves Velasco Forte, Srinivas Narayan, Gerald F Greil, Israel Valverde, Tarique Hussain

**Affiliations:** 1 Guy's and St. Thomas' NHS Foundation Trust, Sutton, United Kingdom; 2 Imaging Sciences and Biomedical Engineering, King's College London, London, United Kingdom

## Background

The combination of 3D printing with CMR images has the potential to allow full, tactile appreciation of the complex of structures that make up a congenital heart abnormality. However, the use of 3D printing is mostly limited to larger teaching hospitals and research centres. Others working in this area have suggested that the technical and medical expertise necessitated by manual image segmentation procedures preclude the translation of this technology into mainstream care. In particular, the laborious nature of existing methods demand an unrealistic amount of the clinician's time to achieve a full segmentation of the relevant structures.

## Methods

A systematic review of the literature, including 124 unique reports on 3D printed models of cardiac anatomy, showed that the majority of reports relied on predominantly manual methods and only considered a single image data set.

**Figure 1:**
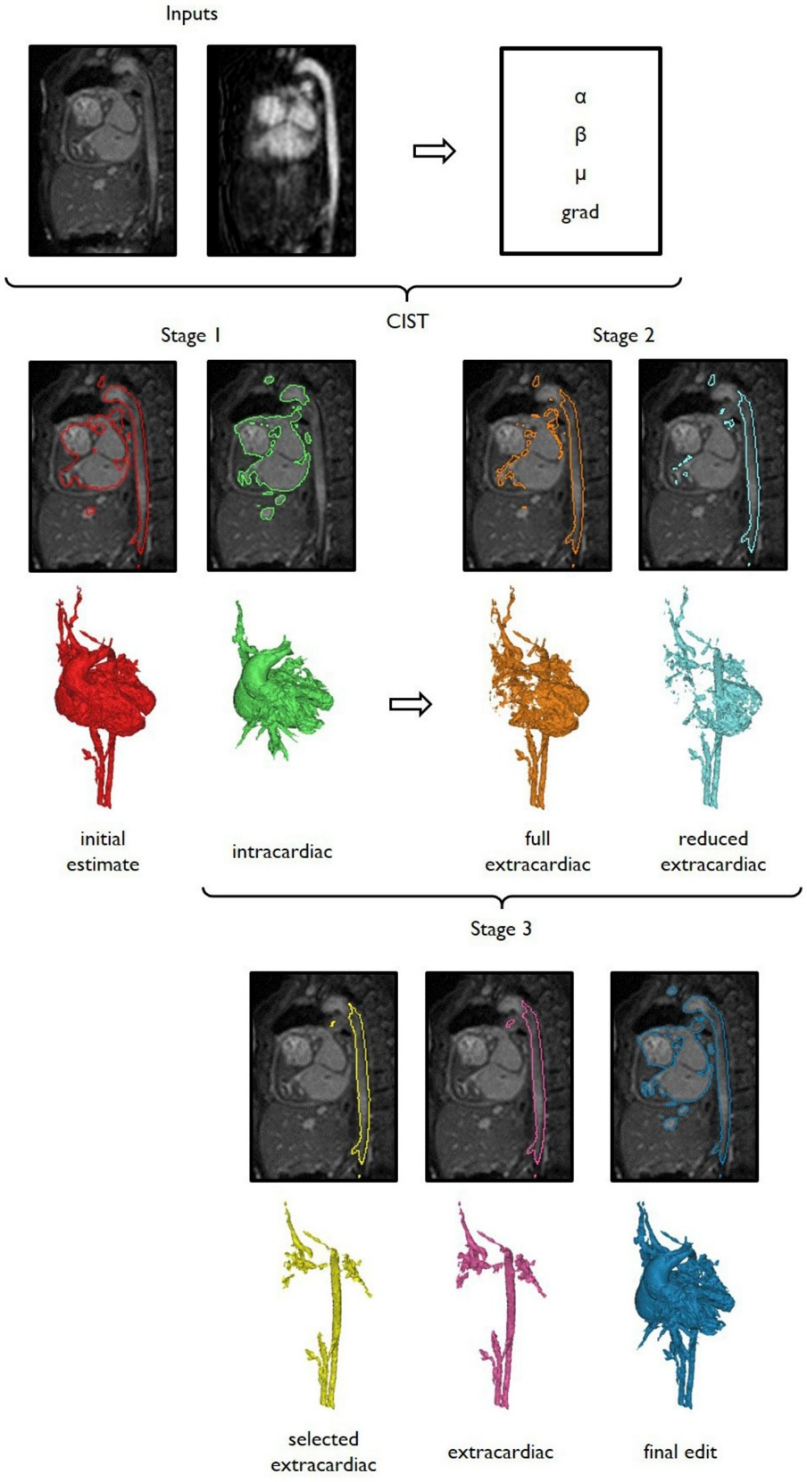
**In Stage 1 of the pipeline, the favourable contrast properties of the gadolinium enhanced MRA are used to generate an initial estimate (red geometry) of the cardiovascular anatomy and guide an automated segmentation of the intracardiac structures (green geometry) in the morphology data**. A hybrid level set procedure, reliant on the parameters alpha, beta, mu and grad is used. In Stage 2, Relevant extracardiac vasculature (orange and light blue geometries) are automatically generated. In Stage 3 the automated segmentation geometries (intracardiac, full and reduced extracardiac masks) are then manually combined using using Boolean and region growing operations. Finally, the slice editing and region growing tools provided by Mimics 18.0 (Materialise NV, Leuven, Belgium, 1992 - 2015) are used to make fianl adjustments to the final segmentation (dark blue geometry).

Based on these observations, a novel image segmentation pipeline has been developed that takes advantage of the complementary information contained within time-resolved angiography (4D MRA) and morphology (3D SSFP) acquisitions (see Figure 1).

The performance of the pipeline was assessed in 6 cases of duct dependent pulmonary atresia with ventricular septal defect. It was compared with manual segmentation alone, both for geometrical consistency and the operator time needed to complete segmentation.

## Results

Automated segmentation delivered a mean Dice Similarity Coefficient (DSC) of 0.87 indicating excellent agreement between novel and manual pipelines. The median operator times for the manual and novel segmentation pipelines were 2 h 0 m (IQR = 2 h 8 m - 1 h 27 m), and 31 m (IQR = 41 m - 24 m), respectively. The median time saving associated with the novel pipeline of 1 h 23 m (IQR = 1 h 35 m - 1 h 1 m) was found to be statistically significant (p = 0.028) using the related samples Wilcoxon signed ranks test.

## Conclusions

The proposed pipeline has demonstrated that information from multi-modal CMR acquisitions can be favourably combined to automate aspects of image segmentation, reducing operator time. The combination of MRA with morphology data is just one example a set of scans that provide complementary information. It is thought that by incorporating further data from the array of images acquired as part of a typical CMR protocol, the segmentation of cardiac structures could become an increasingly automated procedure. Future developments in this area may foster the wider application of 3D printing in the routine care of those with congenital heart disease.

